# Environmental Advocacy by the American College of Emergency Physicians: A Brief History of Climate and Sustainability Resolutions

**DOI:** 10.5811/westjem.53126

**Published:** 2026-02-27

**Authors:** Gayle Galletta, Hillary Irons, Dana Mathew, Marc Futernick, Juliana Chang, Emily Sbiroli, Tushara Surapaneni, David Terca, Niki Thran

**Affiliations:** * University of Massachusetts, Department of Emergency Medicine, Worcester, Massachusetts; † Burrell College of Osteopathic Medicine, Melbourne, Florida; ‡ US Acute Care Solutions LLC, Canton, Ohio; § Northwell Health, Department of Emergency Medicine, Summit, New Jersey; ¶ Royal Hobart Hospital, Department of Emergency Medicine, Tasmania, Australia; || University of Colorado, Department of Emergency Medicine, Boulder, Colorado; # Yale College of Medicine, Department of Emergency Medicine, New Haven, Connecticut; ** Gifford Hospital, Department of Emergency Medicine, Randolph, Vermont

## Abstract

Emergency physicians are on the front lines of climate-driven illness and disaster. Reducing healthcare’s carbon footprint and increasing sustainability can improve planetary and patient health, lower healthcare costs, and boost healthcare job satisfaction. Over the last decade, the American College of Emergency Physicians (ACEP) progressed from early recognition of climate impacts on health to actionable sustainability advocacy. Council resolutions—ACEP’s formal mechanism for policy development—reflects this trajectory, beginning with requests to study climate effects, advancing to coalition engagement, and culminating in operational guidance for reducing emergency department waste and carbon emissions. This paper summarizes the climate and sustainability resolutions presented to the ACEP Council, including brief descriptions and their outcomes. It provides emergency physicians and health system leaders a framework to track and implement ACEP’s sustainability advocacy, with the goal of reducing healthcare’s carbon footprint and improving both planetary and patient health.

## INTRODUCTION

The American College of Emergency Physicians (ACEP) develops policy through its governing body of representatives from state chapters, sections, and affiliated organizations. As of 2025, the ACEP Council is comprised of 436 councilors from 53 state and territorial chapters (based on each chapter’s membership), 40 sections, and representatives from the Emergency Medicine Residents’ Association, the Association of Academic Chairs of Emergency Medicine, the Council of Residency Directors in Emergency Medicine, and the Society for Academic Emergency Medicine.

Any two or more members, chapter, or section can write a resolution that is then presented to the Council. When the Council meets during the two days leading up to the annual ACEP Scientific Assembly, the Council decides to either adopt the resolution, adopt an amended version, not adopt it, or to refer it to the Board of Directors for consideration by a relevant committee, the Council Steering Committee, or the Bylaws Interpretation Committee.

Resolutions adopted by the Council and ratified by the Board become official ACEP policy and guide staff and committee work, educational initiatives, and external advocacy. From 2017–2025, there has been an increasing number of resolutions focused on climate change, environmental stewardship, and sustainability in emergency care. Council resolutions that are explicitly climate- or sustainability-related are listed below with resolution number (and year), title, a summary, the resolved statements, the Council/Board outcome, and actionable items. All resolutions are available on ACEP.org.^1^

## CLIMATE AND SUSTAINABILITY-RELATED ACEP COUNCIL RESOLUTIONS

### Resolution 46 (2017) Impact of Climate Change on Patient Health and Implications for Emergency Medicine

#### Summary

The American College of Emergency Physicians was requested by this resolution to research and develop a new policy statement regarding the impacts of climate change on training, advocacy, preparedness, and patient care in emergency medicine.

#### Resolved

That ACEP research and develop a policy that addresses the impact of climate change on the health and well-being of our patients and use the policy statement to guide future research, training, advocacy preparedness, mitigation practices, and patient care.

#### Outcome

This resolution was initially referred to the Board. After further consideration by that Board, a new policy statement was adopted by ACEP (approved June 2018). It was subsequently revised in September 2024.^2^

#### Actions

Committed ACEP to collaborate with public health agencies and other stakeholders to do the following:

- Raise awareness of the short- and long-term implications of climate change on population health and its effect in the practice of emergency medicine including enhanced patient awareness of medical conditions that may be exacerbated due to weather-related events, applicable mitigation strategies, and early recognition and management of exacerbations.
- Advocate for policies and practices to mitigate and address the effects of climate change on human health, healthcare system preparedness, and public health infrastructure.
- Expand and improve regional surveillance systems of healthcare utilization and emerging diseases associated with climate change, and natural disaster-related injury.
- Collaborate with local government authorities to develop and improve emergency preparedness protocols to coordinate prehospital, intrahospital, and hospital-based emergency services during weather- or natural disaster-related events.
- Advocate for initiatives to reduce the carbon footprint of emergency departments (ED) and their affiliated institutions through energy conservation, healthcare waste reduction and/or recycling, carbon capture initiatives, and purchase contract negotiations that encourage environmental responsibility in the medical product manufacturing and supply chain.
- Advocate for and engage in research examining the effects of climate change on human health, healthcare system access and capabilities, and public health infrastructure as, well as identification of and mitigation for specific vulnerable groups.
- Advocate for research to understand the uneven impact of climate change across different topographies and geographies and create solutions that equitably address gaps in surveillance systems, public health infrastructures, and emergency care response capabilities.
- Encourage cooperation with stakeholders to identify local and regional disaster vulnerabilities and develop specific, regional collaborative action plans, including redundant contingency planning, distribution of resources, and interhospital and interagency cooperation.
- Encourage physician training, independent expertise, and certification in disaster medicine for collaboration with local and regional public health and safety services and other relevant government agencies tasked to address extreme weather events or other natural disasters.

### Resolution 21 (2020): Medical Society Consortium on Climate & Health

#### Summary

Directed ACEP to join the Medical Society Consortium on Climate & Health and support one ACEP representative’s attendance at the annual meeting.

#### Resolved

That ACEP become an official member of the Medical Society Consortium on Climate & Health.

That ACEP support one ACEP member representative by paying registration and travel expenses to attend the Medical Society Consortium on Climate & Health annual meeting starting in 2021.

#### Outcome

Adopted

#### Actions

ACEP became a member of the Consortium and aligned with other medical societies on climate-health advocacy and messaging.^3^

ACEP sends a representative to the annual Consortium meeting.

ACEP disseminates Consortium toolkits and patient-facing materials.

### Board policy action (2018, revised 2024): Impact of Climate Change on Public Health and Implications for Emergency Medicine

#### Summary

Developed in response to Council activity, this policy statement addressed climate impacts on public health and their implications for emergency medicine.^3^

#### Outcome

Approved by the Board.

#### Actions

Provide authoritative language for advocacy at state/federal levels.

Encourage residency programs to include climate health.

Support hospital disaster planning for climate events.

### Resolution 45 (2024) Climate Change Research and Education in Emergency Medicine

#### Summary

Called upon ACEP to encourage and support research on the effects of climate change on health, facilitate data collection on climate-related health emergencies, and support the introduction of climate change curricula in medical schools and residency programs.

#### Resolved

That ACEP encourage and support comprehensive research efforts on the health effects of climate change and the pivotal role of emergency medicine in mitigating and responding to these effects.

That ACEP call for and promote initiatives to facilitate data collection on climate-related health emergencies, such as heat-related illnesses, vector-borne diseases, and extreme weather events, to inform evidence-based interventions, strengthen disaster preparedness, and enhance the capacity to respond effectively to climate change-induced health challenges.

That ACEP support the introduction of curricula that address climate change in medical schools and residency programs.

#### Outcome

Approved. The ACEP policy statement “Impact of Climate Change on Public Health and Implications for Emergency Medicine” partially addressed the resolution.

The Public Health Committee was asked to determine whether changes to the policy statement were needed and any information needed to be included as an adjunct to the policy statement. The third resolution was assigned to the Academic Affairs Committee to recommend that the American Board of Emergency Medicine (ABEM) include climate change in the updated “Model of the Clinical Practice of Emergency Medicine,” which ABEM is in the process of reviewing, as it does every three years.^4^ ACEP selected two representatives to serve on the EM Model Review Task Force.

#### Actions

Research the effects of climate change on health.

Collect data on climate-related health emergencies.

Include climate change and its effect on human health in medical school and curricula.

### Resolution 58 (2024) Reducing Waste in Our Emergency Departments

#### Summary

Called on ACEP to support research and stakeholder collaboration to reduce ED energy consumption, minimize use of disposables, improve recycling and waste segregation, and promote sustainable alternatives when clinically appropriate. This resolution emphasized both environmental stewardship and cost savings.

#### Resolved

That ACEP encourage and support comprehensive research efforts to facilitate data collection of the measurements of ED waste and energy consumption.

That ACEP work with stakeholders, such as hospital administrations, to decrease energy consumption and decrease the amount of hospital waste such as general trash, unused disposables, true plastics, microplastics, and non-recycled glass, as well as biohazard/medical waste.

#### Outcome

Adopted. Assigned to the Public Health Committee to review existing resources and develop a policy statement with input from other stakeholders as identified in second resolved.

#### Actions

Conduct ED waste audits.

Implement recycling/segregation systems.

Partner with vendors for reusable/recyclable products.

Support research on life-cycle analysis and ED waste metrics.

### Resolution 59 (2024) Tap Water Is Sufficient Treatment

#### Summary

Advocated the use of tap water instead of sterile water or saline for wound irrigation, citing evidence that infection rates were comparable.^5^ The resolution aimed to reduce single-use plastic and lower the ED’s carbon footprint.

#### Resolved

That ACEP advocate to transition to hospital tap water in the United States (US) for wound irrigation to decrease the carbon footprint of EDs contributing to global efforts to combat climate change.

That ACEP emphasize the importance of research and education within the emergency medicine community, and to raise awareness of the financial and environmental benefits of tap water for wound irrigation in the United States, highlighting its safety, efficacy, and potential for cost savings.

That ACEP urge policymakers and healthcare administrators to support initiatives that promote sustainable healthcare practices and to advocate for the adoption of tap water for wound irrigation in US emergency settings, aligning with broader efforts to enhance environmental sustainability in healthcare.

#### Outcome

Adopted. ACEP highlighted the timeliness of this resolution following Hurricane Helene, which disrupted sterile water and saline supply chains, and emphasized its potential for significant reductions in plastic waste and carbon emissions.^6^ The first and second resolutions were assigned to the Public Health Committee to review existing resources and consider developing a policy statement. The third resolution was assigned to Advocacy & Practice Affairs staff for advocacy initiatives.

#### Actions

Replace bottled sterile saline and water with potable tap water.

Educate clinicians on evidence base for irrigation.

Track reductions in single-use bottles purchased.

Publicize cost and carbon savings.

### Resolution 61 (2025) Acknowledging and Mitigating the Environmental Impact of Metered-dose Inhalers

#### Summary

Directed ACEP to acknowledge the environmental impact of metered-dose inhalers (MDI), which contain a potent greenhouse gas propellant, and to support efforts to reduce environmental impact through sustainable practices such as advocating for the switch to dry powder inhalers and encouraging proper disposal of MDIs.

#### Resolved

That ACEP acknowledge the environmental impact of MDIs and support efforts to reduce their carbon footprint through sustainable practices.

#### Outcome

Adopted.

#### Actions

Educate clinicians and patients on the high global warming power of hydrofluoroalkane propellants that are used in metered-dose inhalers.^7^

Consider using dry powdered inhalers instead of MDIs when clinically appropriate.

Resist urge to prescribe MDIs to patients without asthma or chronic obstructive pulmonary disease.

Work with health insurers to cover MDI preparations that use a smaller amount of propellant or dry powdered inhalers.

Advocate for manufacturer innovation toward low-global warming power propellants.

Develop recycling programs to incinerate used MDIs, which still contain a large amount of the propellant, even after the medication actuations are empty.

### Resolution 62 (2025) Promoting Environmental Sustainability and Waste Reduction in the ED

#### Summary

This was a broader, chapter-submitted resolution that overlaps with 58 (2024) and called for ACEP to support ED sustainability initiatives including waste reduction, recycling, minimizing low-value interventions, and reducing carbon emissions.

#### Resolved

That ACEP encourage hospitals to implement environmentally responsible practices, including but not limited to proper waste segregation, development of recycling programs, reduction of low-value medical interventions, and the use of sustainable alternatives when clinically appropriate.

#### Outcome

Adopted.

#### Actions

Create an ACEP toolkit for ED sustainability (checklists, best practices).

Develop standardized ED sustainability metrics (waste per patient, carbon per visit).

Share successful models across chapters/sections.

Advocate for federal/state incentives for hospital sustainability programs.

### Resolution 64 (2025) Endorsement of Electronic Discharge Instructions for Patients with Electronic Medical Records

#### Summary

Directed ACEP to encourage EDs to adopt electronic discharge instructions that are Health Insurance Portability and Accountability Act (HIPAA) compliant, patient centered, and environmentally responsible.

#### Resolved

That ACEP endorse the use of electronic discharge instructions for patients with access to electronic medical records and electronic communication capabilities.

That ACEP encourage EDs to adopt policies and technologies that support patient-centered, secure, timely, and environmentally responsible electronic delivery of discharge instructions in compliance with HIPAA regulations.

#### Outcome

Adopted.

#### Actions

Encourage patients to sign up for applications and portals to access their electronic medical records.

Allow patient the option of receiving their discharge instructions electronically, rather than on paper.

## DISCUSSION

The ACEP Council moved from an initial phase of evidence gathering and policy development (2017) to coalition participation (2020), and then to concrete operational interventions at the ED level (2024–2025). This evolution paralleled broader trends in medicine: transitioning from awareness to collaboration and finally to pragmatic, system-level action.

Once adopted and ratified by the Board, resolutions guide ACEP staff and committees in creating policies, educational materials, and advocacy resources. Examples include ACEP’s participation in the Medical Society Consortium on Climate & Health, policy updates, and committee-led initiatives on ED waste reduction and sustainability practices.

Several 2025 submissions built upon or expanded the work initiated in 2024, demonstrating growing member engagement and interest in scaling up ACEP’s sustainability efforts into implementable toolkits, research, and actionable guidance.

Recommendations for emergency physicians, researchers, and advocates:

- Evaluate implementation impact. Track quantifiable outcomes such as ED adoption of tap water irrigation, reductions in single-use plastics, and measurable carbon savings.
- Standardize metrics. Establish common ED sustainability indicators (eg, waste per patient, energy per visit, device lifecycle emissions) to enable benchmarking and aggregated reporting.
- Translate policy into practice. Develop clinical guidance, procurement checklists, and patient communication tools to implement sustainability measures without compromising safety.
- Advance research. Support comparative effectiveness and implementation studies (eg, metered-dose vs dry powder inhalers, electronic discharge instructions adoption rates).
- Join ACEP’s Climate and Sustainability Member Interest Group (MIG). Once the MIG reaches 100 members, it can transition into a Committee with Council representation.
- Engage with your state ACEP chapter. Collaborate with state councilors to strengthen climate-friendly policy initiatives, because grassroots advocacy begins locally.
- Prioritize disaster preparedness. Recognize that EDs act as community safety nets during extreme weather events and support a proactive approach toward procuring resources in anticipation.

## CONCLUSION

Engagement of the American College of Emergency Physicians with climate and sustainability has matured from study and policy formulation to actionable clinical and institutional advocacy. Council resolutions created a roadmap for ACEP to serve as both specialty society and a healthcare-sector leader in climate action. Emergency physicians can advance this work by joining the Climate and Sustainability Member Interest Group, supporting chapter-level advocacy, and contributing research and implementation data. While ACEP does not yet have a formal Climate and Sustainability Committee, the Member Interest Group provides a pathway toward establishing one, ensuring future ACEP climate policy is grounded in both evidence and broad member participation.
